# Supplementary figures and images for: Isolation of SAR11 Marine Bacteria from Cryopreserved Seawater
Source: mSystems. 2020 Dec 22;5(6):e00954-20. doi: 10.1128/mSystems.00954-20 (PMC7762794; doi:10.1128/mSystems.00954-20)

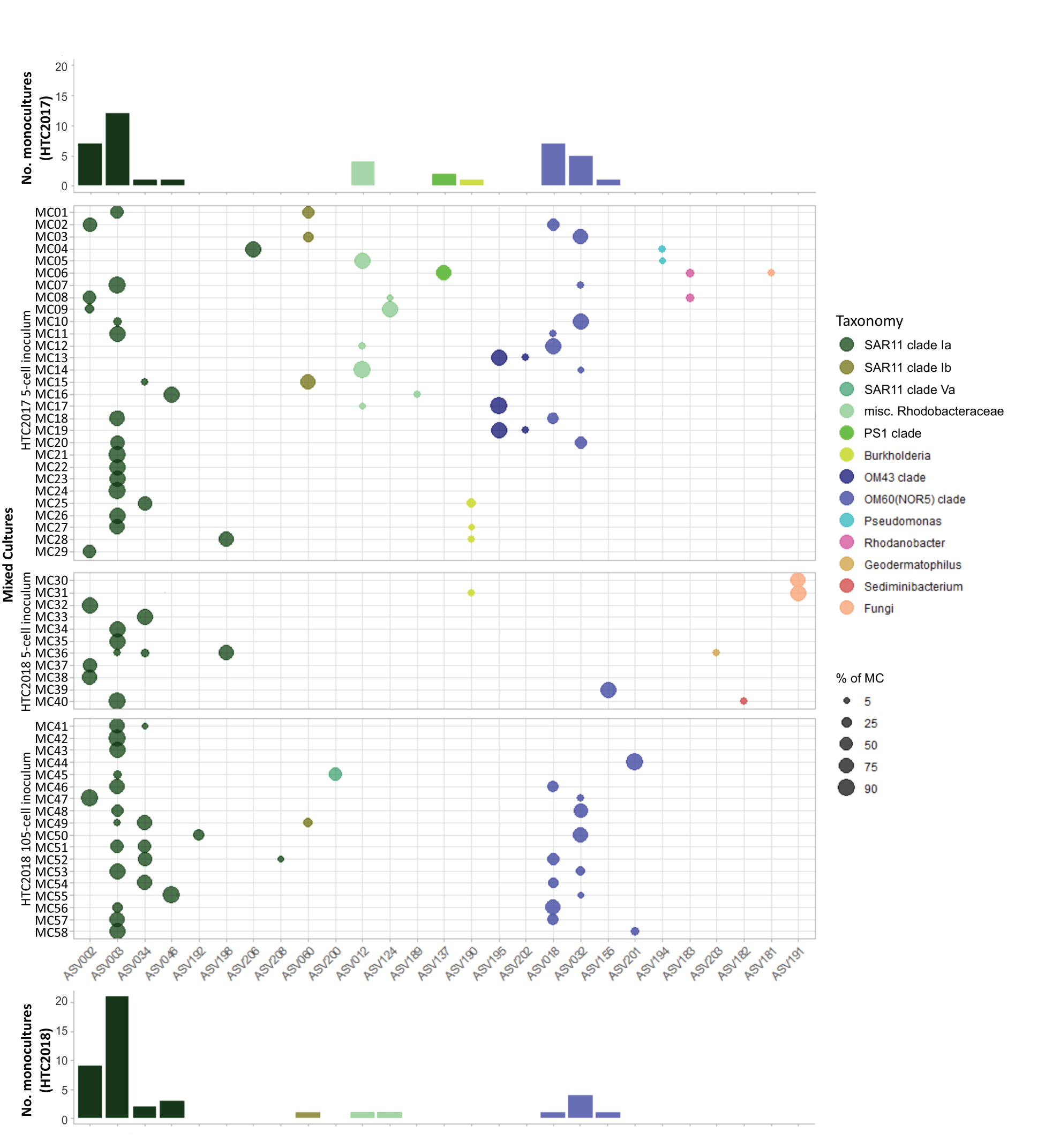

Supplement: FIG S1 [file mSystems.00954-20-sf001.tif]
